# Advancing stereotactic body radiotherapy through off-axis beam optimization to enable safer treatment of multi-isocenter coplanar targets

**DOI:** 10.1016/j.phro.2026.101029

**Published:** 2026-06-29

**Authors:** Mohammad Ali Tajik-Mansoury, Ji N. Lee, Ravneet Kaur, Shailja Patel, Nelly Ju, Khinh Ranh Voong, Reza Farjam

**Affiliations:** a Johns Hopkins University, Department of Radiation Oncology and Molecular Radiation Science, Baltimore, MD, USA; b Johns Hopkins University, Department of Radiation Oncology and Molecular Radiation Science, Washington, DC, USA

**Keywords:** Lung SBRT, Multi coplanar lesions, Off-axis beam optimization

## Abstract

**Background and purpose:**

Oligometastatic lung cancer often requires multiple stereotactic body radiotherapy (SBRT) courses for spatially distinct lesions, increasing the risk of field overlap and side effect. While beam optimization can reduce this risk, posterior lesions remain challenging due to gantry–couch collision constraints. Hence, an off-axis beam optimization framework was developed for posterior lung lesions with direct clinical relevance for coplanar, multi-isocenter SBRT, enabling safer and more efficient dose delivery.

**Materials and methods:**

Twenty-five posterior lung lesions were retrospectively analyzed. For each case, non-optimized, on-axis optimized, and off-axis optimized plans were generated. On-axis optimization preserved central-axis geometry, while off-axis optimization shifted the isocenter toward the midsagittal plane to avoid gantry–couch collision. Dose–volume metrics were compared, and plan efficiency was assessed using isodose line volume (IDLV, 2.5–25 Gy). In selected multi-isocenter cases, overlap between adjacent fields was evaluated.

**Results:**

Optimized beamsets reduced mean lung dose (2.5 ± 1.03 vs 2.3 ± 1 Gy), V_5 Gy_ (445.1 ± 183.7 vs 385.0 ± 168.0 cm^3^), V_10 Gy_ (251.3 ± 123.9 vs 210.6 ± 107.4 cm^3^), and V_20 Gy_ (104.7 ± 63.4 vs 98.2 ± 59.5 cm^3^), with improved organs at risk (OAR) sparing and preserved target coverage. Off-axis plans matched standard plans while eliminating collision risk. In a representative case, IDLV_25 Gy_ and IDLV_20 Gy_ decreased from 406.9 to 270.1 cm^3^ and 668.9 to 502.1 cm^3^, with chest wall V_30 Gy_ reduced from 79.5 to 44.0 cm^3^.

**Conclusions:**

Beam optimization reduces field overlap and OAR dose in multi-lesion SBRT while maintaining plan quality, with off-axis approaches enabling safe, collision-free treatment of posterior lesions.

## Highlights

• Collision-free planning for posterior lung lesions with off-axis beam optimization.
• Decrease in mean lung dose (∼7%), V_5 Gy_ (∼14%), V_10 Gy_ (∼16%) and V_20 Gy_ (∼6%).
• Off-axis plans demonstrated dose–volume metric equivalence to standard coplanar plans.
• Decrease >35% and > 25% in V_25 Gy_ and V_20 Gy_ in a multi-iso coplanar target.
• Decrease >45% in chest wall V_30 Gy_ in a multi-iso coplanar target.

## Graphical abstract

**Figure:**
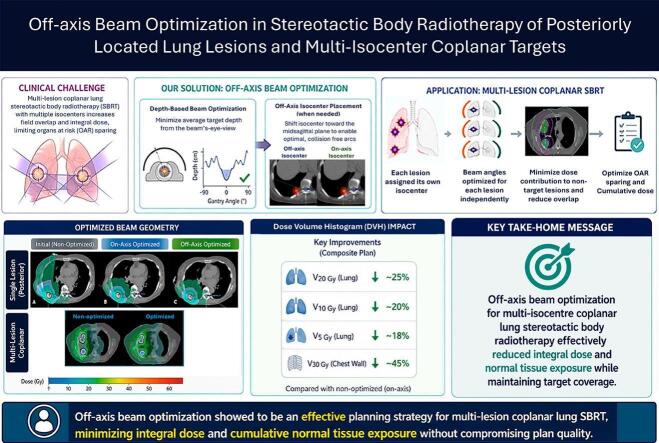

## 1 Introduction

Lung cancer remains the leading cause of cancer-related mortality in the United States, with approximately 248,000 new cases and 130,000 deaths projected in 2026 [1]. Its management is multidisciplinary, including surgery, systemic therapy, and radiotherapy (RT). For medically inoperable early-stage non–small cell lung cancer (NSCLC) and limited oligometastatic disease, stereotactic body radiotherapy (SBRT) is the standard of care, delivering ablative doses with high precision and achieving excellent local control and survival outcomes [2], [3]. However, its effectiveness is fundamentally constrained by the risk of radiation-induced side effect to nearby critical serial (e.g., spinal cord [3], [4], esophagus [4], [5], [6] and proximal bronchial tree [7], [8]) and parallel (e.g., lungs [9], [10], [11], heart [12], [13], [14]) organs within the thorax.

Oligometastatic lung cancer often requires multiple SBRT courses for separate lesions [4], [15], [16], but cumulative organs at risk (OAR) dose becomes a key limitation in re-irradiation. Traditional normal tissue complication probability (NTCP) models (e.g., Lyman [17]), rely on limited dose–volume parameters, restricting retreatment in low-tolerance organs. Emerging machine learning approaches show that the full three-dimensional (3D) dose distribution better capture the risk of adverse events [18], [19] revealing latent patterns not reflected in conventional parameters. Therefore, minimizing normal tissue dose and optimizing Dmax and volumetric constraints is essential to preserve organ function, quality of life, and future treatment options.

Modern delivery techniques such as intensity-modulated radiotherapy (IMRT) and volumetric modulated arc therapy (VMAT) have improved SBRT conformality and OAR sparing [20] but may increase low-dose exposure to normal tissue, highlighting the need for optimized beam configurations. Beam optimization strategies that minimize average target depth from the beam's eye view [21] can reduce integral lung dose, particularly for posterior lesions. However, optimal beam arrangements often require contralateral posterior arcs for such lesions (over 60% of our cases), introducing gantry–couch collision risks that limit clinical use to collision-free but suboptimal geometries, reducing potential planning benefit. To address this, off-axis beam optimization is proposed, hypothesizing that strategic isocenter shifts provide collision-free, optimal beam arrangements for posterior lung lesions without compromising plan quality. However, off-axis beam optimization may introduce uncertainties in both treatment planning and delivery, while multi-isocenter SBRT approaches can suffer from inter-plan field overlap despite improved geometric accuracy for lesions >5 cm apart [22] or with asynchronous motion. Therefore, this study aimed to quantify the planning uncertainties associated with off-axis beam optimization. In addition, we developed and evaluated an optimization strategy to minimize cumulative normal tissue dose while maintaining robust target coverage in complex multi-lesion coplanar SBRT cases treated with independent isocenters [23], [24], [25], [26], [27], [28].

## 2 Materials and methods

### 2.1 Patient selection

A retrospective institutional review board (IRB)-approved study (IRB00449884; consent waived due to de-identified data and retrospective study) included 25 patients with posterior lung lesions treated with SBRT to evaluate off-axis isocenter selection for beam optimization (Table 1). Prescriptions were 10 Gy × 5 (*n* = 23) and 12 Gy × 4 (n = 2), with no dose normalization. All patients were treated on a Versa HD linear accelerator (Elekta, AB, Sweden) with an Agility 160-leaf multileaf collimator (MLC) (5 mm leaf width at isocenter, 1 mm virtual resolution, 40 × 40 cm^2^ field size).

**Table 1:** Characteristics of the patient and lesions used in this study. The average physical depth of the lesion for the initial and optimal beam arrangement is also shown for each lesion. OAD: off-axis distance, PTV: planning target volume, F: Female and M: Male, RUL: right upper lobe, LUL: left upper lobe, RLL: right lower lobe and LLL: left lower lobe, SCCa: Squamous cell carcinoma, NSCLC: non-small cell lung cancer, MPM: malignant peritoneal mesothelioma, SCLC: small cell lung cancer, SPNs: solitary pulmonary nodules, GE, gastrointestinal, Rx: prescription, ${\bar{d}}_{\mathrm{ph}}$: Average physical depth.

No.	F/M	Lobe	OAD (cm)	Primary Diagnosis		Dose (Rx)	PTV (cm^3^)	${\bar{d}}_{\mathrm{ph}}$(cm)	
					TNM Staging			Initial beam-set	Optimal beam-set
1	M	RLL	6.2	NSCLC	T1cN0M0	10 * 5	71.5	15.4	11.7
2	M	RLL	4.4	MPM	Metastasis	10 * 5	31.8	13.7	9.7
3	M	RLL	7.7	Sarcoma	Metastasis	10 * 5	45.9	10	6
4	F	RUL	7.4	NSCLC	T1cN0M0	10 * 5	53.6	17.4	15.5
5	M	LLL	7.0	NSCLC	T1cN0M0	10 * 5	20.8	13.2	7.6
6	F	RLL	3.5	SCCa of epiglottis	Metastasis	10 * 5	16	12.6	7.4
7	F	RLL	10.5	Lung Cancer	T1N0M0	10 * 5	28.2	8.4	5.9
8	F	RUL	5.6	NSCLC	T1bN0M0	10 * 5	30.5	10.9	7.7
9	M	RLL	5.5	SPNs	Solitary nodule	10 * 5	25.1	15.3	13.3
10	F	RLL	6.7	SPNs	Solitary nodule	10 * 5	9.7	17.4	12.2
11	F	LLL	9.4	SPNs	Solitary nodule	10 * 5	11.5	9.3	8.1
12	F	LUL	6.2	Lung Cancer	T1N0M0	10 * 5	49.4	9.1	8.1
13	M	LUL	5.3	SPNs	Solitary nodule	10 * 5	25.3	11.5	10.3
14	F	LUL	8.5	Lung Cancer	T1N0M0	10 * 5	29.3	12.3	10.7
15	F	LUL	8.5	Lung Cancer	T1N0M0	12 * 4	16.8	8.2	6.9
16	F	RLL	7.9	NSCLC	Metastasis	10 * 5	5.1	7.3	4
17	M	LLL	13.0	N/A	Metastasis	10 * 5	16.3	8	4.5
18	F	LLL	5.2	Lung Cancer	T1N0M0	10 * 5	17.2	8.5	6
19	F	RLL	5.7	Lung Cancer	T1N0M0	10 * 5	35.7	11.4	6.1
20	M	RLL	9.3	Lung Cancer	T2aN0M0	10 * 5	53.3	9	6.3
21	F	RLL	10.7	Lung Cancer	T1N0M0	10 * 5	19.7	4.5	3.6
22	M	RLL	5.5	SPNs	Solitary nodule	10 * 5	36.1	16.9	7.7
23	M	LLL	8.8	Lung Cancer	T1N0M0	10 * 5	7.9	8.5	4.1
24	F	RLL	6.3	Lung Cancer	T1N0M0	12 * 4	20	12.1	11.3
25	F	RLL	3.8	NSCLC	T1N0M0	10 * 5	8	14.2	10.1

### 2.2 Off-axis beam optimization

The beam optimization approach described in our previous work has been followed [21]. In brief, the optimal beam arrangement for VMAT-based SBRT was a set of half-arcs determined by maximizing the therapeutic gain (TG) through minimizing the average lesion depth relative to the beam's eye view (BEV), as described below:

(1) $$\mathrm{TG}\left(b_{\theta }\right)\propto \frac{1}{d_{\mathrm{ph},\theta }\left(\mathrm{iso}\right)}$$

(2) $$\mathrm{TG}\left(\mathrm{Arc}\left(\theta_{\mathrm{start}},\theta_{\mathrm{stop}}\right)\right)\propto \frac{\theta_{\mathrm{Stop}}-\theta_{\mathrm{Start}}}{\int_{\theta_{\mathrm{start}}}^{\theta_{\mathrm{stop}}}d_{\mathrm{ph},\theta }\left(\mathrm{iso}\right)}$$

**Fig. 1:**
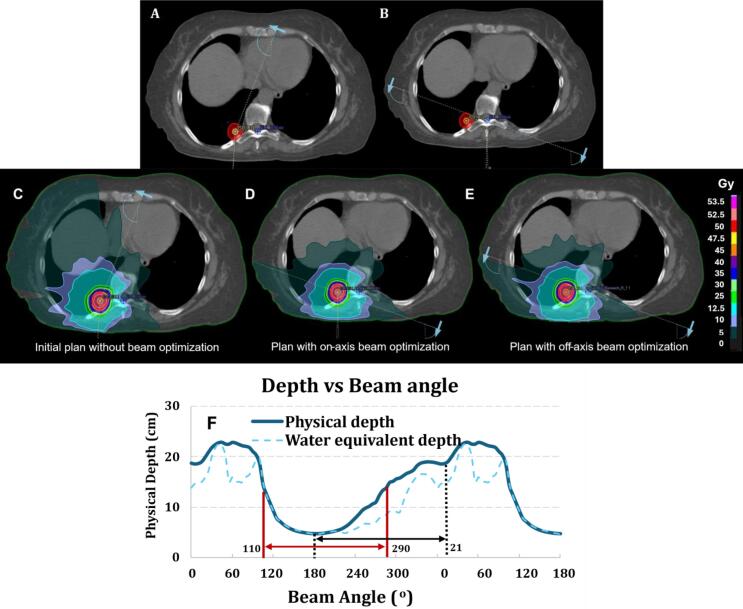
A representative example of off-axis isocenter selection for a posterior lung lesion to ensure a collision-free beam arrangement. Beam arrangement (A) and Plan (C) without beam path optimization (gantry angles 21°–181°). Optimized beam arrangement (gantry angles 110°–290°) with the corresponding off-axis isocenter location (B). Plans with optimized on-axis (D) and off-axis (E) isocenter selection. (F) Lesion depth vs. beam angle curve for the same lesion. Both on-axis (yellow) and off-axis (blue) isocenter locations are illustrated. (For interpretation of the references to colour in this figure legend, the reader is referred to the web version of this article.)

In these equations, *b*_θ_ denotes the beam at the angle θ, and *d*_ph,θ_ indicates the physical depth of the lesion with respect to the BEV at that angle. For each target, a plot of physical depth versus beam angle was generated using an in-house script to provide the 180^o^ arc-set with minimum area under the curve as the optimal beam set, Fig. 1C.

As shown in Fig. 1B, optimized beamsets for posterior lesions often require contralateral posterior arcs, increasing gantry–couch collision risk or necessitating couch shifts and additional imaging time. To address this, off-axis beam optimization was applied to posterior lesions to eliminate collision risk by shifting the isocenter within the mid-sagittal plane while keeping axial and coronal positions unchanged (Fig. 1A–B). For this study, a central position was used to provide consistent distance sampling and reduce selection bias, whereas in clinical practice the off-axis point is chosen near the planning target volume (PTV) boundary while avoiding collision risk.

### 2.3 Treatment planning

Each case was replanned using two configurations, on-axis and off-axis beam optimization, to assess the impact of isocenter selection for posterior lung lesions. Plans were compared against the original clinical plans using an in-house script [21] that generated physical depth–beam angle maps to define optimal beam sets (Table 1, all 25 lesions). A “superior” plan was defined as one that improved target coverage, conformity index, and OAR sparing without degrading any individual metric. In addition, the total monitor units (MU) had to be lower than the original clinical plan. This MU requirement was imposed to ensure that improvements were not simply due to increased optimization effort or plan complexity but reflected a more efficient beam arrangement. The same evaluation criteria were applied consistently to both on-axis and off-axis replans. The same clinical objectives as in the previous study were used [21], with plan conformity evaluated using Radiation Therapy Oncology Group (RTOG) conformity indices [29]. Planning was performed in RayStation version 2023B (RaySearch laboratories, Sweden) [30]. Clinical plans used two partial arcs (clockwise and counterclockwise) with 2° gantry spacing and 6 MV FFF beams. In the revised plans, each arc was replaced by two arcs (lateral and contralateral posterior) with identical parameters (Fig. 1B). Dosimetrists were blinded to study details during original plan creation.

### 2.4 Plan evaluation

To assess the impact of off-axis beam optimization, both on-axis and off-axis optimized plans were compared with the original non-optimized plans to determine whether optimization improved plan quality. A direct comparison between the two optimized cohorts was also performed. In addition to standard clinical objectives [21], isodose line volumes (IDLV 25, 20, 15, 10, 5, 2.5, and 1 Gy) were calculated to quantify dose spread. Dose-volume metrics (Table 2) and isodose volumes were analyzed to evaluate differences in dose distribution. Statistical significance between on-axis and off-axis cohorts was assessed using paired Student's *t*-tests.

**Table 2:** Statistical comparison of dose–volume indices between initial plans and optimized plans with on-axis and off-axis isocenter placement. The off-axis cohort (Off) was compared with both the initial (In) and on-axis (On) cohorts. *P*-values are reported for each metric, with statistical significance defined as *P* < 0.05.

Structure	Example shown in Fig. 1			Initial (In)	On axis (On)	Off-axis (off)	In vs off (*p-*Value)	On vs Off (*p-*Value)
	Initial	On-axis	Off-axis					
GTV: V_100_	100.0	100.0	100.0	100 ± 0	100 ± 0	100 ± 0	N/A	N/A
PTV: V_100_	95.5	95.5	95.5	95.7 ± 1	95.8 ± 1	95.8 ± 1	0.0095	0.78
Gradient Index	4.2	3.9	3.9	4.3 ± 0.6	4.1 ± 0.5	4.1 ± 0.5	0.00038	0.85
Coverage Quality	0.9	0.9	0.9	0.9 ± 0	0.9 ± 0	0.9 ± 0	0.0086	0.12
D_max_ at 2 cm/Rx	42.5	40.4	40.7	47.8 ± 5.7	45.9 ± 6.7	46 ± 6.6	0.0024	0.62
PTV: Mean	61.30	61.29	62.14	60.74 ± 1.20	61.03 ± 13.3	60.93 ± 12	0.00048	0.43
IDLV_25 Gy_ (cm^3^)	66.9	62.0	62.2	113.4 ± 64.2	110.6 ± 64.2	110.7 ± 64.4	6.05E-05	0.89
IDLV_20 Gy_ (cm^3^)	103.5	96.1	96.6	178.1 ± 104.4	172.8 ± 104	172.8 ± 104.1	0.0081	0.97
IDLV_15 Gy_ (cm^3^)	182.6	168.5	169.8	327.1 ± 216.9	312.7 ± 205.8	312.2 ± 203.2	0.0098	0.67
IDLV_10 Gy_ (cm^3^)	403.3	343.5	347.9	731.2 ± 515.7	642 ± 439.1	648.4 ± 450.6	5.48E-05	0.12
IDLV_5 Gy_ (cm^3^)	1415.8	884.7	892.3	1653.7 ± 889	1368.7 ± 768.9	1376 ± 777.3	2.79E-07	0.041
MLD (Gy)	2.3	2.02	2.04	2.5 ± 1.03	2.34 ± 0.98	2.36 ± 1	1.38E-08	0.0038
Lungs, V_20 Gy_ (cm^3^)	57.8	50.7	50.2	104.7 ± 63.4	98 ± 59	98.2 ± 59.5	0.00062	0.42
Lungs, V_10 Gy_ (cm^3^)	184.9	147.0	147.7	251.3 ± 123.9	210.6 ± 107.4	212 ± 109.3	8.13E-09	0.084
Lungs, V_5 Gy_ (cm^3^)	334.4	269.5	273.0	445.1 ± 183.7	382.1 ± 166.6	385 ± 168	7.15E-06	0.042
CW, V_30 Gy_ (cm^3^)	18.3	17.0	17.4	15.9 ± 11.6	15.3 ± 11.5	15.2 ± 11.5	0.001	0.19
CW, V_45 Gy_ (cm^3^)	9.3	8.4	6.2	4.2 ± 4.3	4 ± 4.2	3.9 ± 4.2	0.024	0.24
Cord Max (Gy)	15.66	14.41	14.56	10.60 ± 4.43	9.98 ± 4.27	9.85 ± 4.32	1.41E-05	0.234
Skin Max (Gy)	15.93	15.27	15.13	17.80 ± 5.55	17.33 ± 5.28	17.29 ± 5.24	0.0003	0.334
Pericardium Max (Gy)	9.31	6.31	6.47	6.47 ± 4.26	5.7 ± 3.94	5.71 ± 3.94	0.00027	0.904
Airway Max (Gy)	3.8	3.0	3.0	6.95 ± 7.73	61.9 ± 6.90	6.15 ± 6.96	0.0014	0.384
Esophagus Max (Gy)	5.76	5.03	5.00	8.82 ± 3.68	79.7 ± 3.32	79.9 ± 3.51	0.00033	0.864
Total MU	3118.0	2442.0	2619.0	3127.1 ± 719.5	2790.4 ± 598.7	2872.8 ± 673.2	0.00023	0.0744

### 2.5 Clinical implementation

*Collision assessment for posterior lesions:* Collision clearance for posterior lung SBRT was assessed using a low-resolution volumetric scout at simulation, which also defined the isocenter. An in-house script evaluated gantry–patient clearance, with collisions deemed unlikely if anatomy fitted within an ∼80 cm diameter cylinder (including a 5 cm margin) centered at the isocenter. When feasible, a contralateral posterior arc was used; otherwise, a lateral half-arc with minimal depth was delivered. For multi-target coplanar cases with a posterior lesion, an off-axis isocenter was employed as described below.

*Technical basis for SBRT of coplanar lesions:* After assessing breathing patterns and target motion, the team selected single- or multi-isocenter strategies for coplanar lesions. In multi-isocenter cases, each lesion centroid served as its own isocenter with independently optimized beam geometry. Posterior lesions underwent collision checks, with isocenters shifted toward the midsagittal plane if needed. Plans were then optimized to minimize non-target dose, reduce field overlap, improve OAR sparing, and limit cumulative beam exposure.

## 3 Results

Target volumes ranged from 5.11 to 71.52 cm^3^ (27.4 ± 16.8 cm^3^). As shown in Table 1, beam optimization reduced lesion treatment depth by 28.4% ± 14.4 (6.4–54.3%). Fig. 1 demonstrates a representative posterior case with ∼41% depth reduction. Table 2 shows that both optimized plans significantly improved OAR sparing and target coverage versus the initial plan, with the on-axis approach yielding a slightly, but not significantly, better plan than the off-axis approach.

**Fig. 2:**
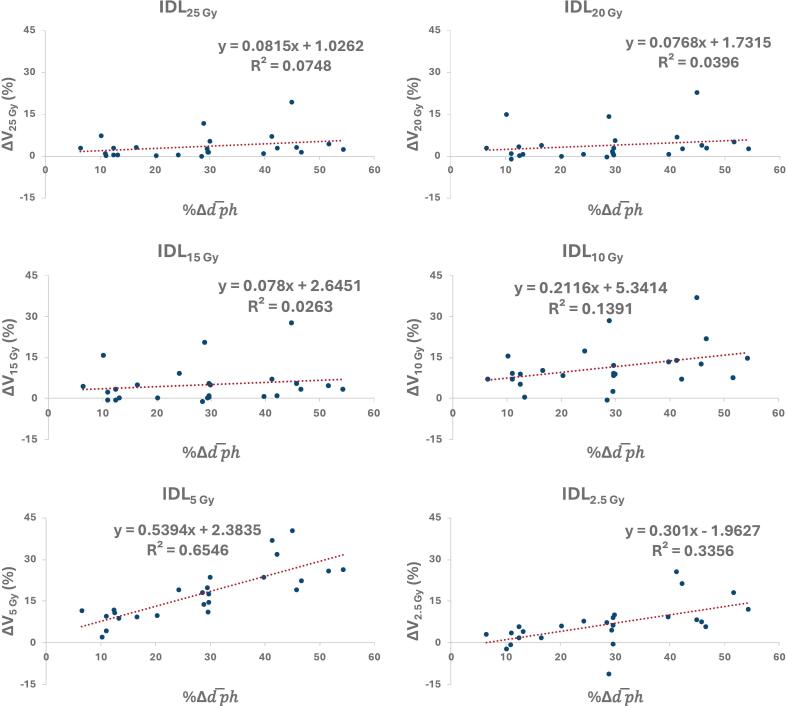
Relationship between changes in lesion depth relative to the beam's eye view and the corresponding variation in isodose line volumes. As average of the treatment depth decreases, the overall treatment impact on the normal tissue diminishes, with lower isodose line volumes showing a more pronounced reduction because of decreased beam penetration depth.

As shown in Fig. 2, decreasing treatment depth (in BEV) reduced the dose impact on surrounding normal tissue. The magnitude of reduction was greater at lower isodose levels, whereas smaller changes were observed at higher dose levels. It was found that the largest mean ± SD volume reduction after optimization occurred consistently at the 5 Gy isodose line (Supplementary Material A, Fig. S1).

**Fig. 3:**
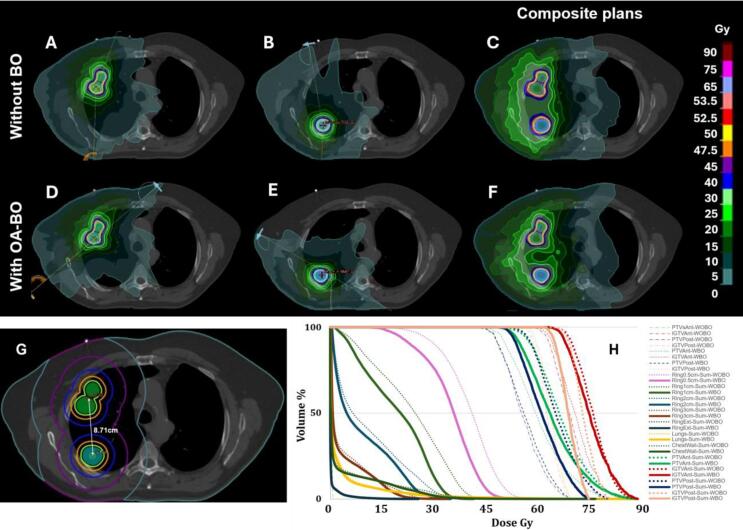
Example of coplanar targets treated with off-axis beam optimization (OA-BO). (A–B) Original plans without beam optimization. (D–E) Plans with OA-BO. Composite dose distributions and dose volume histograms (DVH) for both scenarios are also shown (C, F, and H). For the posterior lesion, the off-axis isocenter was positioned near the PTV while resolving clearance issues. As shown in H, OA-BO resulted in a substantial reduction in the volumes enclosed by the 25 Gy, 20 Gy, and 10 Gy isodose lines. (G) Lesion contours along with a series of abutting ring structures (0.5 cm, 1 cm, 2 cm, and 3 cm in thickness), plus an external ring, all generated concentrically around the combined target volume.

Fig. 3(A–F) demonstrates that off-axis beam optimization reduced isodose volumes and overall dose spillage for both individual coplanar lesions and their composite plan. Dose–volume histogram analysis (DVH) further confirmed improved normal tissue sparing in the optimized and composite plans relative to the original plans while preserving target coverage, Fig. 3(G–H). Notably, beam optimization reduced the 25 Gy and 20 Gy isodose volumes significantly, leading to lower lung V_20 Gy_ and V_10 Gy_. Also, high-dose reduction yielded ∼45% decrease in chest wall V_30 Gy_. The reduced 25 Gy volume also improved D_95%_, bringing it closer to the prescription dose. Consistent with these findings, Table 3 shows reductions in dose to all evaluated OARs. Additional coplanar cases are provided in Supplementary Material B, Figs. S2–S4.

**Table 3:** Dose–volume comparison of coplanar lesions with and without beam optimization is shown in Fig. 3. Beam optimization reduced field overlaps and decreased inter-plan dose contribution, resulting in >40% reduction in posterior lesion overdose, 30% reduction in IDLV_25 Gy_, 12% reduction in mean lung dose, and 45% reduction in chest wall V_30 Gy_.

Structure	Ant (Rx: 10 Gy * 5)		Post (Rx: 12 Gy * 5)		Composite plan			
					Initial		OA-BO	
	Initial	BO	Initial	OA-BO	Ant	Post	Ant	Post
GTV: V_100_	100.0	100.0	100.0	100.0	100	100	100	100
PTV: D_95_ (Gy)	50.0	50	48	48.1	56.8	57.4	54.04	53.2
Gradient Index	4.7	4.6	5.1	4.6	N/A	N/A	N/A	N/A
Coverage Quality	0.9	0.9	0.9	0.9	0.9	1	0.9	1
D_max_ at 2 cm/Rx	48.7	48.4	46.0	45.9	73.4	77.4	70.3	69.0
PTV: Mean	60.84	61.06	56.44	57	67.61	66.44	65.21	62.17
IDLV25 Gy (cm^3^)	97.1	87.4	62.1	55.1	406.9		270.1	
IDLV20 Gy (cm^3^)	159.1	149.1	99.9	88.3	668.9		502.1	
IDLV10 Gy (cm^3^)	735.1	627.5	452.1	414.6	1479.9		1422.5	
IDLV5 Gy (cm^3^)	1510.5	1312.6	1248.6	949.8	2365.1		2252.0	
MLD (Gy)	1.8	1.6	1.4	1.2	3.2		2.8	
Lungs, V20 (cm^3^)	101.8	93.7	67.3	59.9	274.4		249.7	
Lungs, V10 (cm^3^)	269.0	229.6	186.6	144.5	526.6		478.9	
Lungs, V5 (cm^3^)	541.9	467.0	421.4	317.4	836.3		752.3	
CW, V30 Gy (cm^3^)	11.7	10.1	6.1	5.4	79.5		44.0	
CW, V45 Gy (cm^3^)	2.1	1.8	0.3	0.2	7.7		3.6	
Cord Max (Gy)	5.47	5.20	5.92	5.84	11.07		10.64	
Skin Max (Gy)	12.48	12.45	13.71	12.63	21.93		18.04	
Pericardium Max (Gy)	8.9	6.7	4.1	3.3	1.2		9.5	
Airway Max (Gy)	8.63	8.21	6.45	6.21	14.66		13.97	
Esophagus Max (Gy)	7.1	6.76	5.58	5.06	11.90		11.55	
Total MU	3944.0	3742.0	3261.0	3230.0	N/A		N/A	

## 4 Discussion

In this study, an off-axis beam optimization framework was developed to enable safer and more efficient treatment of posterior lesions, where beam access was frequently limited by couch–gantry collision constraints, occurring in more than 60% of such cases at our institution. The proposed framework achieved dose–volume metrics comparable to standard planning approaches while providing immediate applicability to complex clinical scenarios, including multi-isocenter coplanar treatments.

Consistent with prior findings, off-axis beam optimization (OA-BO) improved plan quality, reduced lung V_20 Gy_, V_10 Gy_, V_5 Gy_, mean lung dose, and other OAR doses by minimizing lesion depth (Table 2). This effect was further reflected in decreased low-dose isodose volumes (e.g., IDLV_10 Gy_ and IDLV_5 Gy_), indicating smaller overall plan volumes (Fig. 3 and Fig. S1). At our institution, the standard approach is to use on-axis optimized beamsets for posterior lesions when no couch–gantry collision is anticipated. Collision clearance is verified during planning using the simulation scout image, a low-resolution volumetric dataset that includes patient anatomy and immobilization devices. However, many patients do not pass this collision assessment, limiting the broader applicability of conventional beam optimization.

The findings of this study also suggested that off-axis isocenter placement produced a plan comparable to on-axis optimization while reducing couch–gantry collision risk. Dose–volume comparisons showed that beam optimization consistently improved PTV coverage, conformity, and OAR sparing regardless of isocenter position. Although differences between on- and off-axis optimized plans were not statistically significant, on-axis plans showed a slight trend toward greater reductions in isodose line volumes (Table 2, Fig. S1), supporting safe use of OA-BO when needed. Figs. 3 and S1 also demonstrate a correlation between beam's-eye-view depth reduction and decreased IDLVs, mostly pronounced at lower dose levels and peaking at IDLV_5 Gy_. This correlation diminished at very low doses (e.g., IDLV_2.5 Gy_ and IDLV_1 Gy_) due to body-contour truncation, which could underestimate true dose spread in an unbounded medium.

The primary use of off-axis beam optimization at our institution is for coplanar lesions treated with separate isocenters. These cases benefit most from independent beam optimization while naturally avoiding couch–gantry collision due to geometric separation of targets. However, a key limitation of this study is its reliance on treatment planning metrics without comprehensive experimental validation. Although the results demonstrated feasibility and safe implementation within the treatment planning system, the lack of systematic verification (e.g., patient-specific quality assurance (QA) or phantom studies) limits confirmation of delivery accuracy. Accordingly, although no formal guidelines currently restrict the use of off-axis isocenters in SBRT, this technique has not yet been adopted for routine clinical use at our institution pending comprehensive validation of its delivery accuracy and safety. Ongoing work is focused on quantifying the residual uncertainties associated with off-axis isocenter placement and evaluating their potential clinical impact.

Supplementary cases supported the consistency of treatment planning trends in multi-isocenter scenarios; however, the main conclusions were based on a limited number of representative examples, including the highlighted case of multi-isocenter coplanar lesions. As a result, generalizability remains limited, and larger cohorts with measurement-based validation are needed to confirm robustness and clinical applicability. Despite this limitation, off-axis optimization is selectively applied in multi-isocenter coplanar cases due to its clear planning benefit at our institution. In our practice, lesions separated by >5 cm are typically treated with separate isocenters to avoid coverage loss [22], with final decisions made on a case by case basis. Because each field contributes dose to the other target, off-axis optimization can reduce inter-field effects; any small underdosage risk from one plan is generally compensated by overlapping coverage from the complementary plan. For example, in Fig. 3 and Table 3, D_95%_ for the posterior lesion decreased from ∼57.3 Gy to ∼53.2 Gy (vs. 50 Gy prescription), while combined plans maintained adequate coverage with additional safety margin. Importantly, this approach also improved OAR sparing, particularly for the chest wall and lung. Chest wall V_30 Gy_ was reduced by approximately 45% (79.5 to 44.0 cm^3^), potentially decreasing the risk of treatment-related adverse events [31], with concurrent reductions in V_25 Gy_ and V_20 Gy_, also reflected in DVHs of the ring structures (Fig. 3, Table 3). These gains are clinically relevant given the prevalence and anatomical variability of lung cancer presentations in oligometastatic disease [32]. Finally, due to geometric variability and the limited number of multi-isocenter cases, results were presented using representative examples without loss of generality. Ongoing work is focused on expanding the dataset to better define the clinical utility of this framework.

In conclusion, this study evaluated off-axis beam optimization for SBRT of posterior lung lesions. The results showed that it can extend depth-based beam angle optimization, enabling improved treatment geometry in cases limited by couch–gantry collision risk. For single posterior targets, it produced robust plans, although further delivery validation is needed before broad clinical adoption; quantifying the delivery uncertainty to establish comprehensive guidelines for off-axis beam optimization is underway. For multi-isocenter coplanar lesions, however, the technique was immediately translatable. It reduced inter-lesion dose overlap and cumulative OAR exposure, supporting safer and more effective multi-target SBRT. More broadly, off-axis delivery aligns with emerging platforms such as Magnetic Resonance Imaging-guided Linear Accelerator (MRI-Linac) [33], Positron Emission Tomography Linear Accelerator (PET-Linac) [34], and Tomotherapy [35], which are well suited to implement this strategy. In contrast, couch-rotation (couch-kick) approaches [36], [37] can improve beam access but may increase low-dose spread, introduce isocenter shifts, and add setup uncertainty due to limited post-rotation imaging, limiting compatibility with modern image-guided workflows. Off-axis beam optimization therefore provides a practical and safer alternative in these scenarios.

## CRediT authorship contribution statement

**Mohammad Ali Tajik-Mansoury:** Writing – review & editing, Writing – original draft, Validation, Software, Resources, Methodology, Investigation, Formal analysis, Data curation, Conceptualization. **Ji N. Lee:** Writing – review & editing, Writing – original draft, Resources, Investigation, Data curation, Conceptualization. **Ravneet Kaur:** Writing – review & editing, Investigation, Data curation, Conceptualization. **Shailja Patel:** Writing – review & editing, Investigation, Data curation, Conceptualization. **Nelly Ju:** Writing – review & editing, Investigation, Data curation, Conceptualization. **Khinh Ranh Voong:** Writing – review & editing, Validation, Resources, Investigation, Conceptualization. **Reza Farjam:** Writing – review & editing, Writing – original draft, Visualization, Validation, Supervision, Software, Resources, Project administration, Methodology, Investigation, Formal analysis, Data curation, Conceptualization.

## Declaration of competing interest

The authors declare that they have no known competing financial interests or personal relationships that could have appeared to influence the work reported in this paper.

## References

[1] Siegel R.L., Kratzer T.B., Wagle N.S., Sung H., Jemal A. (2026). Cancer statistics, 2026. CA Cancer J Clin.

[2] Stanic S., Paulus R., Timmerman R.D., Michalski J.M., Barriger R.B., Bezjak A. (2014). No clinically significant changes in pulmonary function following stereotactic body radiation therapy for early- stage peripheral non-small cell lung cancer: an analysis of RTOG 0236. Int J Radiat Oncol Biol Phys.

[3] Chang J.Y., Senan S., Paul M.A., Mehran R.J., Louie A.V., Balter P. (2015). Stereotactic ablative radiotherapy versus lobectomy for operable stage I non-small-cell lung cancer: a pooled analysis of two randomised trials. Lancet Oncol.

[4] Bradley J.D., Paulus R., Komaki R., Masters G., Blumenschein G., Schild S. (2015). Standard-dose versus high-dose conformal radiotherapy with concurrent and consolidation carboplatin plus paclitaxel with or without cetuximab for patients with stage IIIA or IIIB non-small-cell lung cancer (RTOG 0617): a randomised, two-by-two factorial phase 3 study. Lancet Oncol.

[5] Senan S., Brade A., Wang L.-H., Vansteenkiste J., Dakhil S., Biesma B. (2016). PROCLAIM: randomized phase III trial of pemetrexed-cisplatin or etoposide-cisplatin plus thoracic radiation therapy followed by consolidation chemotherapy in locally advanced nonsquamous non-small-cell lung cancer. J Clin Oncol.

[6] Chen C., Uyterlinde W., Sonke J.-J., de Bois J., van den Heuvel M., Belderbos J. (2013). Severe late esophagus toxicity in NSCLC patients treated with IMRT and concurrent chemotherapy. Radiother Oncol.

[7] Cannon D.M., Mehta M.P., Adkison J.B., Khuntia D., Traynor A.M., Tomé W.A. (2013). Dose-limiting toxicity after hypofractionated dose-escalated radiotherapy in non-small-cell lung cancer. J Clin Oncol.

[8] Timmerman R., McGarry R., Yiannoutsos C., Papiez L., Tudor K., DeLuca J. (2006). Excessive toxicity when treating central tumors in a phase II study of stereotactic body radiation therapy for medically inoperable early-stage lung cancer. J Clin Oncol.

[9] Roy S., Salerno K.E., Citrin D.E. (2021). Biology of radiation-induced lung injury. Semin Radiat Oncol.

[10] Arroyo-Hernández M., Maldonado F., Lozano-Ruiz F., Muñoz-Montaño W., Nuñez-Baez M., Arrieta O. (2021). Radiation-induced lung injury: current evidence. BMC Pulm Med.

[11] Palma D.A., Senan S., Tsujino K., Barriger R.B., Rengan R., Moreno M. (2013). Predicting radiation pneumonitis after chemoradiation therapy for lung cancer: an international individual patient data meta-analysis. Int J Radiat Oncol Biol Phys.

[12] Stam B., Peulen H., Guckenberger M., Mantel F., Hope A., Werner-Wasik M. (2017). Dose to heart substructures is associated with non-cancer death after SBRT in stage I-II NSCLC patients. Radiother Oncol.

[13] Stam B., van der Bijl E., van Diessen J., Rossi M.M.G., Tijhuis A., Belderbos J.S.A. (2017). Heart dose associated with overall survival in locally advanced NSCLC patients treated with hypofractionated chemoradiotherapy. Radiother Oncol.

[14] Banfill K., Giuliani M., Aznar M., Franks K., McWilliam A., Schmitt M. (2021). Cardiac toxicity of thoracic radiotherapy: existing evidence and future directions. J Thorac Oncol.

[15] Machtay M., Bae K., Movsas B., Paulus R., Gore E.M., Komaki R. (2012). Higher biologically effective dose of radiotherapy is associated with improved outcomes for locally advanced non-small cell lung carcinoma treated with chemoradiation: an analysis of the Radiation Therapy Oncology Group. Int J Radiat Oncol Biol Phys.

[16] Aupérin A., Le Péchoux C., Rolland E., Curran W.J., Furuse K., Fournel P. (2010). Meta-analysis of concomitant versus sequential radiochemotherapy in locally advanced non-small-cell lung cancer. J Clin Oncol.

[17] Lyman J.T. (1985). Complication probability as assessed from dose-volume histograms. Radiat Res.

[18] Cui S., Ten Haken R.K., El Naqa I. (2021). Integrating multiomics information in deep learning architectures for joint actuarial outcome prediction in non-small cell lung cancer patients after radiation therapy. Int J Radiat Oncol Biol Phys.

[19] Dudas D., Saghand P.G., Dilling T.J., Perez B.A., Rosenberg S.A., El Naqa I. (2024). Deep learning-guided dosimetry for mitigating local failure of patients with non-small cell lung Cancer receiving stereotactic body radiation therapy. Int J Radiat Oncol Biol Phys.

[20] Lee J., Dean C., Patel R., Webster G., Eaton D.J. (2019). Multi-center evaluation of dose conformity in stereotactic body radiotherapy. Phys Imaging Radiat Oncol.

[21] Hooshangnejad H., Lee J., Bell L., Hales R.K., Voong K.R., Han-Oh S. (2025). Quantitative beam optimization for radiotherapy of peripheral lung lesions: a pilot study in stereotactic body radiotherapy. J Appl Clin Med Phys.

[22] Critchfield L.C., Bernard M.E., Randall M.E., McGarry R.C., Pokhrel D. (2020). Risk of target coverage loss for stereotactic body radiotherapy treatment of synchronous lung lesions via single-isocenter volumetric modulated arc therapy. J Appl Clin Med Phys.

[23] van Timmeren J.E., Ehrbar S., Chamberlain M., Mayinger M., Hoogeman M.S., Andratschke N. (2022). Single-isocenter versus multiple-isocenters for multiple lung metastases: evaluation of lung dose. Radiother Oncol.

[24] Sanford L., Molloy J., Kumar S., Randall M., McGarry R., Pokhrel D. (2019). Evaluation of plan quality and treatment efficiency for single-isocenter/two-lesion lung stereotactic body radiation therapy. J Appl Clin Med Phys.

[25] Trager M., Salama J., Yin F.-F., Adamson J. (2017). SBRT treatment of multiple extracranial oligometastases using a single isocenter with distinct optimizations. J Radiosurg SBRT.

[26] Pokhrel D., Sanford L., Halfman M., Molloy J. (2019). Potential reduction of lung dose via VMAT with jaw tracking in the treatment of single-isocenter/two-lesion lung SBRT. J Appl Clin Med Phys.

[27] Sanford L., Pokhrel D. (2019). Improving treatment efficiency via photon optimizer (PO) MLC algorithm for synchronous single-isocenter/multiple-lesions VMAT lung SBRT. J Appl Clin Med Phys.

[28] Quan K., Xu K.M., Lalonde R., Horne Z.D., Bernard M.E., C McCoy (2015). Treatment plan technique and quality for single-isocenter stereotactic ablative radiotherapy of multiple lung lesions with volumetric-modulated arc therapy or intensity-modulated radiosurgery. Front Oncol.

[29] Feuvret L., Noël G., Mazeron J.-J., Bey P. (2006). Conformity index: a review. Int J Radiat Oncol Biol Phys.

[30] Bodensteiner D. (2018). RayStation: external beam treatment planning system. Med Dosim.

[31] Andolino D.L., Forquer J.A., Henderson M.A., Barriger R.B., Shapiro R.H., Brabham J.G. (2011). Chest wall toxicity after stereotactic body radiotherapy for malignant lesions of the lung and liver. Int J Radiat Oncol Biol Phys.

[32] Couñago F., Luna J., Guerrero L.L., Vaquero B., Guillén-Sacoto M.C., González-Merino T. (2019). Management of oligometastatic non-small cell lung cancer patients: current controversies and future directions. World J Clin Oncol.

[33] Liney G.P., Whelan B., Oborn B., Barton M., Keall P. (2018). MRI-linear accelerator radiotherapy systems. Clin Oncol.

[34] Oderinde O.M., Shirvani S.M., Olcott P.D., Kuduvalli G., Mazin S., Larkin D. (2021). The technical design and concept of a PET/CT linac for biology-guided radiotherapy. Clin Transl Radiat Oncol.

[35] Welsh J.S. (2008). Helical tomotherapy: a fascinating technological concept that has matured into clinical reality. Technol Cancer Res Treat.

[36] Kei T., Luca K., Kayode O., Higgins K.A., Bradley J.D., Shelton J.W. (2025). Improving lung stereotactic body radiation therapy dose conformity using a simple noncoplanar volumetric modulated arc therapy technique. Pract Radiat Oncol.

[37] Ye W., Wang H., Wei Z., Zhang W., Yu C., Zhang D. (2024). Dosimetric investigation of couch rotation angles in non-coplanar VMAT plans for lung cancer SBRT. Front Oncol.

