# Supplementary material for: Advancing stereotactic body radiotherapy through off-axis beam optimization to enable safer treatment of multi-isocenter coplanar targets
Source: Phys Imaging Radiat Oncol. 2026 Jun 29;40:101029. doi: 10.1016/j.phro.2026.101029 (PMC13380209; doi:10.1016/j.phro.2026.101029)
Supplement: Supplementary file 1 — Supplementary material [file mmc1.pdf]

Supplementary Materials A. Changes in various isodose line volume after beam optimization

Fig. S1 presents the statistical analysis (Mean ± STD) of volume reductions for various isodose line volumes following beam optimization. Consistent with Fig. 2, the beam optimization yields the greatest average reduction at the 5 Gy isodose line.

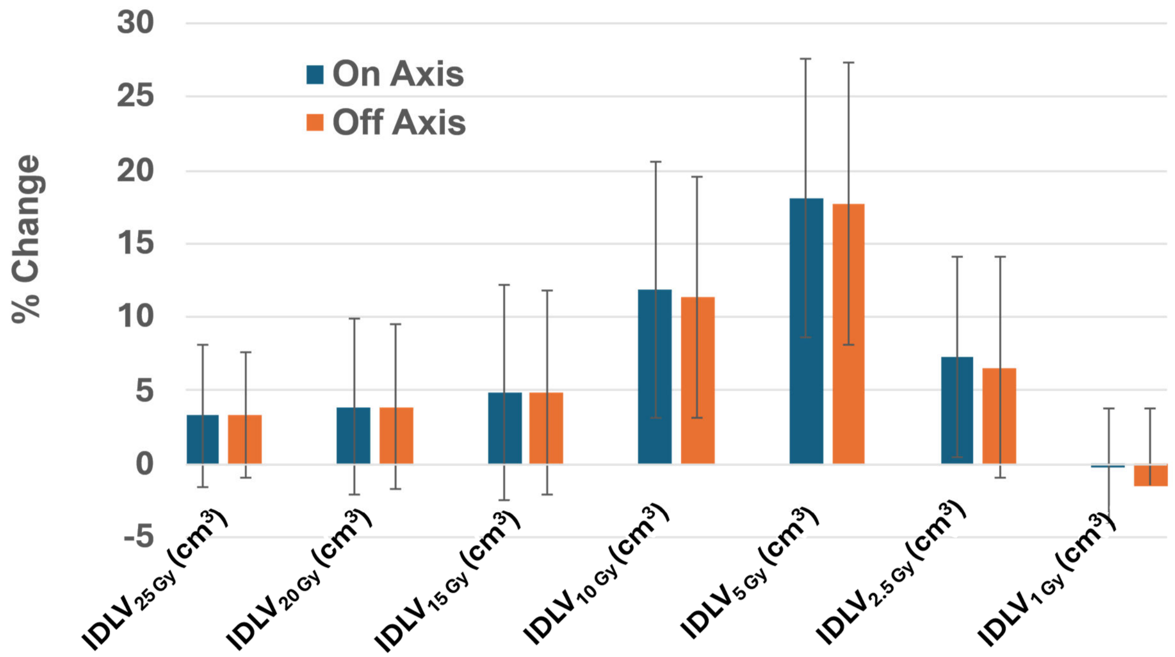

Fig S1. Changes in various isodose line volumes following beam optimization with both on-axis and off-axis isocenter placement. As shown, the on-axis approach provides slightly greater—but not statistically significant—dose sparing compared to the off-axis approach, indicating that both optimized plans outperform the non-optimized plan.

Supplementary Materials B. Examples of co-planar lesions planned with Off-axis beam optimization

In Figs S2-S4, three more representative coplanar cases planned using both optimized and non-optimized beamsets are presented. For each case, a dosimetrist independently generated clinically acceptable plans with each approach. Composite plans were then created for both optimized and non-optimized beamsets. Because the optimized beamset reduced the volume encompassed by various isodose lines, we quantified and compared the volumes of selected isodose levels ($IDLV_{20\ Gy}$, $IDLV_{10\ Gy}$, $IDLV_{5\ Gy}$) across these examples. Additionally, mean lung dose (MLD) and lung $V_{5\ Gy}$, $V_{10\ Gy}$, and $V_{20\ Gy}$ are reported for both scenarios.

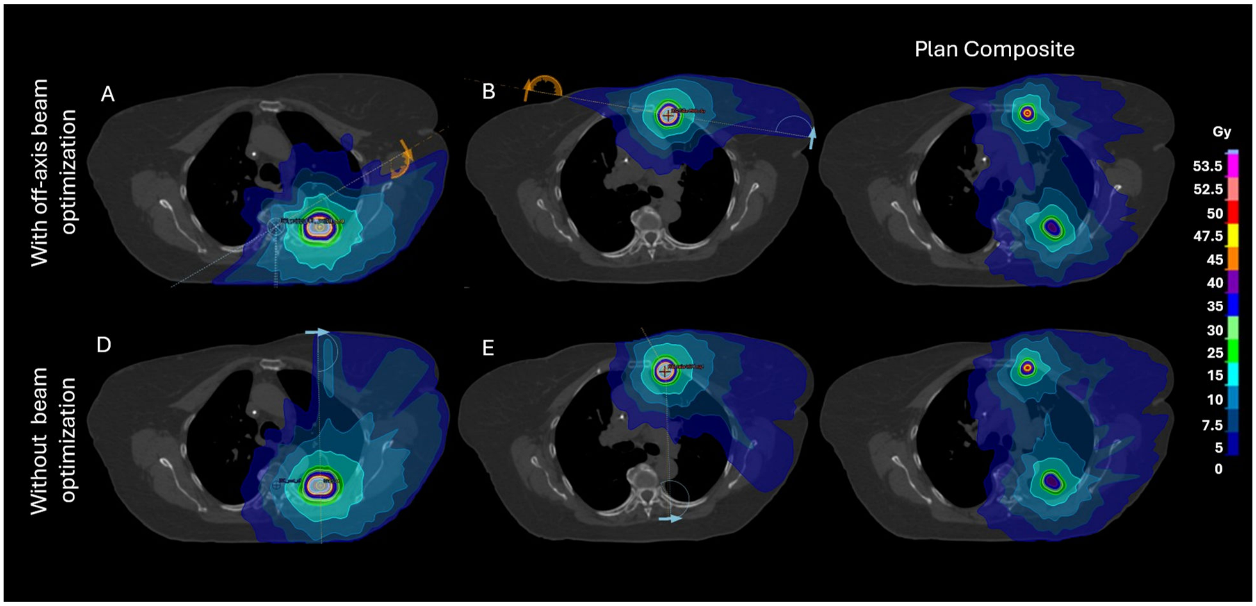

Fig. S2. A patient with anterior and posterior lesions located at left upper lobe planned using optimized (A, B) and non-optimized (D, E) beam set. Composite plans are also shown. For plans with non-optimal beamset, $IDLV_{20\,Gy}$ = 109.9 cm^3, $IDLV_{10\,Gy}$ = 564.9 cm^3, $IDLV_{5\,Gy}$ = 1955.3 cm^3 and for plans with optimal beamset, $IDLV_{20\,Gy}$ = 103.2 cm^3, $IDLV_{10\,Gy}$ = 474.3 cm^3, $IDLV_{5\,Gy}$ = 1450 cm^3. For plans with non-optimal beamset, MLD = 2.91 Gy, $V_{5\,Gy}$ = 456.2 cm^3, $V_{10\,Gy}$ = 196 cm^3, $V_{20\,Gy}$ = 68 cm^3. For plans with optimal beamset, MLD = 2.64 Gy, $V_{5\,Gy}$ = 398.8 cm^3, $V_{10\,Gy}$ = 142.2 cm^3, $V_{20\,Gy}$ = 61.8 cm^3.

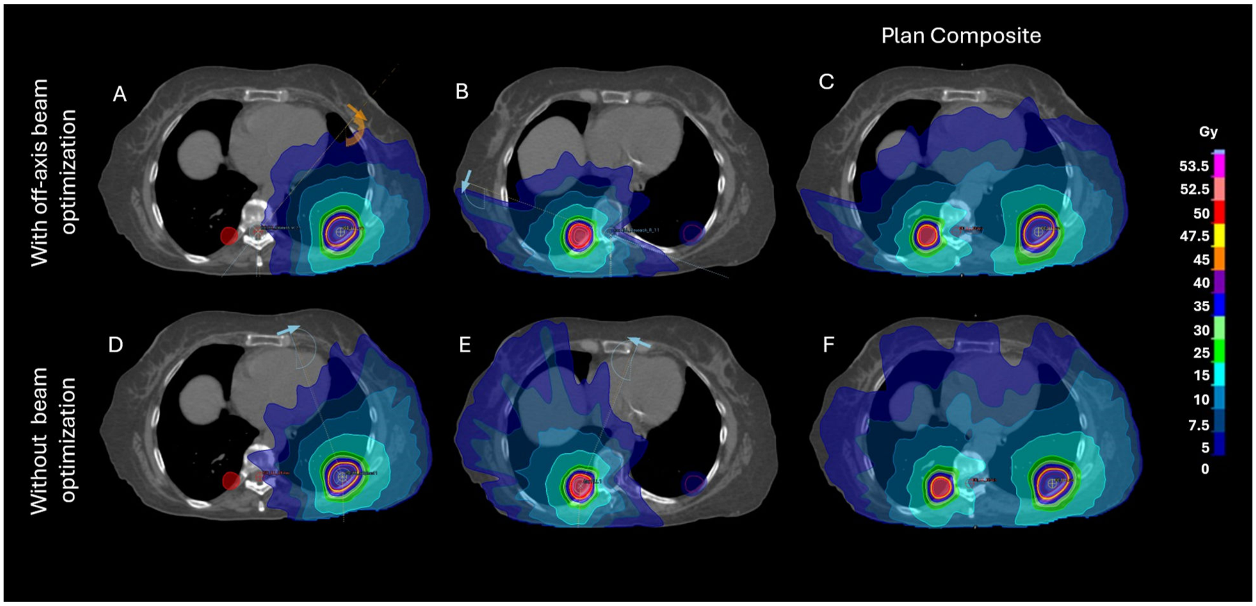

Fig. S3. A patient with right and left lesions located at lower lobe planned using optimized (A, B) and non-optimized (D, E) beam set. Composite plans are also shown. For plans with non-optimal beamset, $IDLV_{20\,Gy}$ = 280.5 cm^3, $IDLV_{10\,Gy}$ = 1298.1 cm^3, $IDLV_{5\,Gy}$ = 3006.8 cm^3 and for plans with optimal beamset, $IDLV_{20\,Gy}$ = 272.1 cm^3, $IDLV_{10\,Gy}$ = 1044.1 cm^3, $IDLV_{5\,Gy}$ = 2266.4 cm^3. For plans with non-optimal beamset, MLD = 5 Gy, $V_{5\,Gy}$ = 836.3 cm^3, $V_{10\,Gy}$ = 448.2 cm^3, $V_{20\,Gy}$ = 148.5 cm^3. For plans with optimal beamset, MLD = 4.59 Gy, $V_{5\,Gy}$ = 749.5 cm^3, $V_{10\,Gy}$ = 373.1 cm^3, $V_{20\,Gy}$ = 132.7 cm^3.

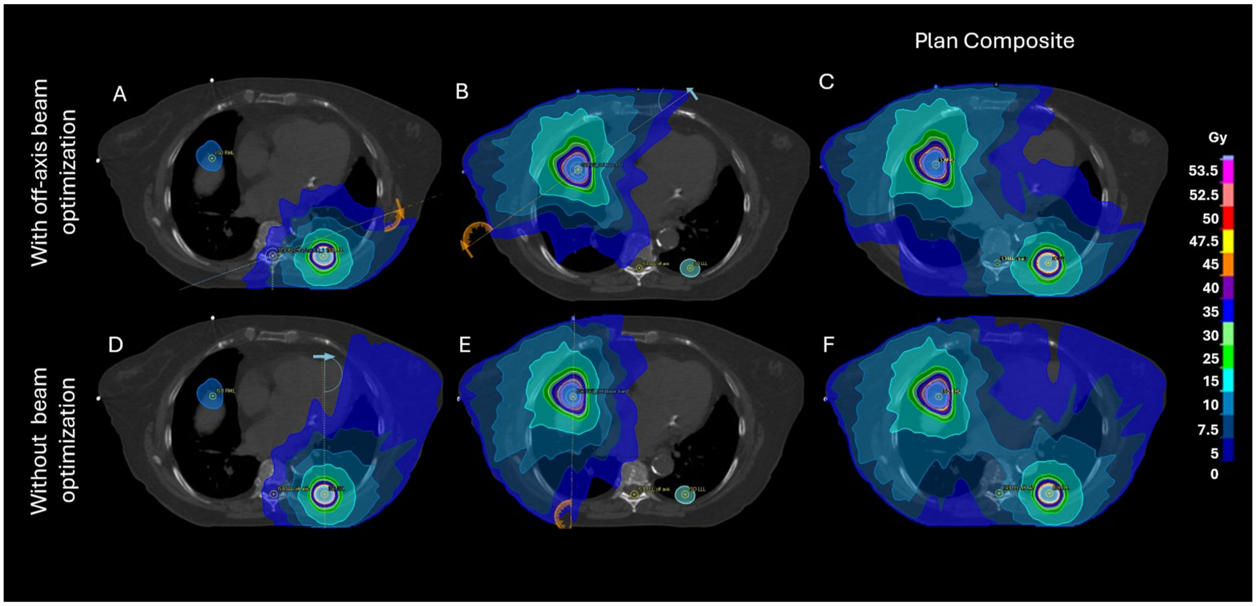

Fig S4. A patient with posterior and anterior lesions located at left lower lobe and right medial lobe planned using optimized (A, B) and non-optimized (D, E) beam set. Composite plans are also shown. For plans with non-optimal beamset, $IDLV_{20\ Gy}$ = 248 cm^3, $IDLV_{10\ Gy}$ = 1239.1 cm^3, $IDLV_{5\ Gy}$ = 3362.9 cm^3 and for plans with optimal beamset, $IDLV_{20\ Gy}$ = 246 cm^3, $IDLV_{10\ Gy}$ = 1119 cm^3, $IDLV_{5\ Gy}$ = 2679 cm^3. For plans with non-optimal beamset, MLD = 4.91 Gy, $V_{5\ Gy}$ = 797.6 cm^3, $V_{10\ Gy}$ = 449.2 cm^3, $V_{20\ Gy}$ = 152.8 cm^3. For plans with optimal beamset, MLD = 4.56 Gy, $V_{5\ Gy}$ = 738.8 cm^3, $V_{10\ Gy}$ = 347 cm^3, $V_{20\ Gy}$ = 150.9 cm^3.
